# Supplementary material for: Three-Dimensional Quantitative Structure–Activity Relationship Study of Transient Receptor Potential Vanilloid 1 Channel Antagonists Reveals Potential for Drug Design Purposes
Source: Int J Mol Sci. 2024 Jul 21;25(14):7951. doi: 10.3390/ijms25147951 (PMC11276937; doi:10.3390/ijms25147951)
Supplement: Supplementary file 1 [file ijms-25-07951-s001.zip › ijms-3108471-supplementary.pdf]

Table S1. Compounds 3a-z of the training set.

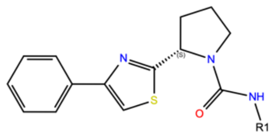

Compounds	R1	Inhibition rate (%)
3a	o-Br-phenyl	38
3b	m-Br-phenyl	23
3c	p-Br-phenyl	47
3d	o-Cl-phenyl	50
3e	m-Cl-phenyl	45
3f	p-Cl-phenyl	64
3g	o-F-phenyl	54
3h	m-F-phenyl	44
3i	p-F-phenyl	83
3j	o-CF3-phenyl	50
3k	m-CF3-phenyl	36
3l	p-CF3-phenyl	65
3m	o-OMe-phenyl	22
3n	p-OMe-phenyl	41
3o	o-iPr-phenyl	59
3p	m-iPr-phenyl	39
3q	o-NO2-phenyl	25
3r	4-Cl-2-NO2-phenyl	56
3s	2,5-diCl-phenyl	35
3t	2,4,6-triCl-phenyl	27
3u	2,4,6-triMe-phenyl	44
3v	2,4-diMe-phenyl	49
3w	2,5-diMe-phenyl	75
3x	2-chloropyridin-3-	49
3y	isoquinoline-5-	77
3z	p-tBu-phenyl	46

Table S2. Compounds 4a-i, 5a-s, 6 of the training set.

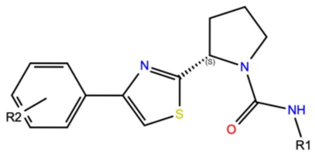

Compounds	R1	R2	Inhibition rate (%)
4a	o-F-phenyl	3-F	32
4b	m-F-phenyl	3-F	26
4c	p-F-phenyl	3-F	48
4d	o-CF3-phenyl	3-F	32

4e	m-CF3-phenyl	3-F	16
4f	p-CF3-phenyl	3-F	57
4g	phenyl	3-F	33
4h	2-chloropyridin-3-	3-F	47
4i	isoquinoline-5-	3-F	71
5 a	o-Br-phenyl	4-F	32
5b	m-Br-phenyl	4-F	23
5c	p-Br-phenyl	4-F	42
5d	o-Cl-phenyl	4-F	61
5e	m-Cl-phenyl	4-F	48
5f	p-Cl-phenyl	4-F	77
5g	o-F-phenyl	4-F	49
5h	m-F-phenyl	4-F	28
5i	p-F-phenyl	4-F	71
5j	o-CF3-phenyl	4-F	65
5k	m-CF3-phenyl	4-F	47
5l	p-CF3-phenyl	4-F	68
5m	o-OMe-phenyl	4-F	73
5n	p-OMe-phenyl	4-F	5
5o	o-iPr-phenyl	4-F	17
5p	o-NO2-phenyl	4-F	33
5q	2-chloropyridin-3-	4-F	25
5r	isoquinoline-5-	4-F	77
5s	p-tBu-phenyl	4-F	40
6	p-CF3-phenyl	4-Me	42

Table S3. Compounds 7a-aa of the training set.

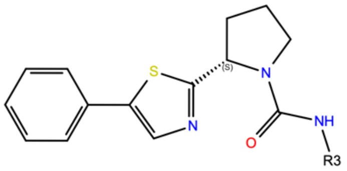

Compounds	R3	Inhibition rate (%)
7a	o-Br-phenyl	55
7b	m-Br-phenyl	49
7c	p-Br-phenyl	39
7d	o-Cl-phenyl	27
7e	m-Cl-phenyl	44
7f	p-Cl-phenyl	46
7g	o-F-phenyl	42
7h	m-F-phenyl	43
7i	p-F-phenyl	39
7j	o-CF3-phenyl	44
7k	m-CF3-phenyl	71

7l	p-CF3-phenyl	34
7m	o-OMe-phenyl	64
7n	p-OMe-phenyl	63
7o	3,4-diOMe-phenyl	67
7p	o-iPr-phenyl	81
7q	m-iPr-phenyl	88
7r	2-NO2-phenyl	78
7s	4-Cl-2-NO2-phenyl	61
7t	4-Me-2-NO2-phenyl	77
7u	4-Me-3-Cl-phenyl	50
7v	2,4,6-triCl-phenyl	36
7w	2,4,6-triMe-phenyl	54
7x	2,4-diMe-phenyl	58
7y	2,5-diMe-phenyl	56
7z	2-chloropyridin-3-	40
7aa	isoquinoline-5-	60
